# Caprine herpesvirus 2-associated malignant catarrhal fever of captive sika deer (*Cervus nippon*) in an intensive management system

**DOI:** 10.1186/s12917-018-1365-8

**Published:** 2018-02-01

**Authors:** Hongwei Zhu, Qingrong Huang, Xiaoliang Hu, Wenhui Chu, Jianlong Zhang, Linlin Jiang, Xin Yu, Xingxiao Zhang, Shipeng Cheng

**Affiliations:** 1 School of Life Sciences, Ludong University, No. 186 Hongqi Middle Rd., Zhifu District, Yantai, 264025 China; 2 Harbin Veterinary Research Institute, Chinese Academy of Agricultural Sciences, Harbin, 150069 China; 3 Institute of Special Economic Animal and Plant Sciences, Chinese Academy of Agricultural Sciences, No. 4899 Juye St., Jingyue District, Changchun, 130112 China

**Keywords:** Sika deer, *Cervus nippon*, Malignant catarrhal fever, CpHV-2, Lameness, Aerosol route transmission

## Abstract

**Background:**

Caprine herpesvirus 2 (CpHV-2) infection usually induces chronic malignant catarrhal fever (MCF) in sika deer (*Cervus nippon*), with the primary signs of weight loss, dermatitis and alopecia.

**Case presentation:**

Here, we report a case of CpHV-2-associated acute MCF in a sika deer herd raised in an intensive management system distant to the reservoir goats. Affected deer developed clinical signs of high fever (41 °C) followed by nasal discharge and lameness. Severe lesions of hemorrhage, necrosis and infiltration of lymphoid cells could readily be observed in the lung, kidney, heart valves and subcutaneous tissue surrounding a tendon. Etiologically, identical CpHV-2 specific DNA sequences were detected in peripheral blood lymphocyte (PBL) from the affected deer and reservoir goats.

**Conclusion:**

In summary, domestic goats were the reservoir of the CpHV-2, which is the causative agent of the outbreak of MCF in the three hinds. The disease was probably transmitted via aerosol infection. In addition, necrosis and inflammation in subcutaneous tissue surrounding a tendon was the reason for lameness. Therefore, MCF should be put into a differential diagnostic list when similar disease occurs in sika deer herds.

**Electronic supplementary material:**

The online version of this article (10.1186/s12917-018-1365-8) contains supplementary material, which is available to authorized users.

## Background

Malignant catarrhal fever (MCF) is a lymphoproliferative disease, caused by a group of closely related ruminant viruses within the genus *Macavirus*, in the subfamily *Gammaherpesvirinae* [1]. The disease is characterized as sporadic with low incidence and high mortality in bison, cattle, buffalo, deer and antelope [2, 3]. Etiologically, alcelaphine herpesvirus 1 (AlHV-1) and ovine herpesvirus 2 (OvHV-2) are the most common causative agents. Subclinically infected wildebeest and domestic sheep act as natural reservoirs, causing wildebeest-associated MCF (WA-MCF) and sheep-associated MCF (SA-MCF), respectively. In general, AIHV-1 causes MCF syndromes in ruminant wild animals in Africa and zoological animals worldwide, whereas OvHV-2 is more prevalent in domestic sheep, causing MCF in most regions of the world. In addition to AlHV-1 and OvHV-2, caprine herpesvirus 2 (CpHV-2), ibex-MCFV, MCFV–white-tailed deer (WTD), and alcelaphine herpesvirus 2 (AlHV-2) have been reported to be pathogenic under natural conditions [4].

Upon infection, different clinical manifestations could be observed in the host depending on the affected organs. In most cases, early clinical signs of the disease usually begin with high fever and depression, followed by catarrhal nasal discharges, mucosal ulcers and/or cloudy corneas. Dysentery or bloody diarrhea is common in most cases [2, 5]. Oculonasal discharge could be an indication of the so-called head-and-eye form of MCF, whereas the intestinal tract form, the central nervous system (CNS) form and the cutaneous form of the disease are easily confused with other diseases.

Pathologically, heart, brain, lung and kidney are the most easily affected organs, although lesions may be found in any organ with different severities and frequencies. Characteristic lymphoid cell accumulation and infiltration in various tissues can be readily observed during microscopic investigation. Lymphocytic vasculitis is a typical histological lesion of the disease [5, 6].

A wide range of animals in the family *Cervidae* are susceptible to malignant catarrhal fever virus (MCFV) infections. These deer species include sika deer (*Cervus nippon*), white-tailed deer (*Odocoileus virginianus*), Père David’s deer (*Elaphurus davidiansus*), sambar deer (*Cervus unicolor*), mule deer (*Odocoileus hemionus*), moose (*Alces alces*), red deer (*Cervus elaphus*), red brocket deer (*Mazama americana*), elk (*Cervus canadensis*), rusa deer (*Cervus timorensis*), Chinese water deer (*Hydropotes inermis*) and axis deer (*Axis axis*) [4, 7–13]. In general, sika deer seem highly susceptible to OvHV-2 [14–16]. Peracute cases with sudden death and acute cases with hemorrhagic diarrhea are well documented and common in our experience of clinical investigation. CpHV-2 infection in deer, on the contrary, is rarely encountered and usually produces mild and chronic clinical signs such as weight loss, dermal inflammation and alopecia. The pathogenic significance of CpHV-2 was reported shortly after its recognition: four outbreaks of CpHV-2-associated MCF in sika deer and white-tailed deer in Florida, Arizona, Minnesota and Texas in the US were described with the primary signs of weight loss and alopecia [17–19]. Before the causative agent was identified, a case of white-tail deer infection was reported in a North American zoo as well [20, 21]. Later, in Europe, CpHV-2-induced MCFs were also documented in deer species in Norway and UK [12, 22]. In this report, we described, for the first time in China, the outbreak of an acute MCF caused by CpHV-2 from domestic goats 1.0 km in distance. Significant differences were found with respect to clinical signs and gross and histological lesions, compared with the previous CpHV-2-induced MCFs in sika deer.

## Case presentation

**Fig. 1:**
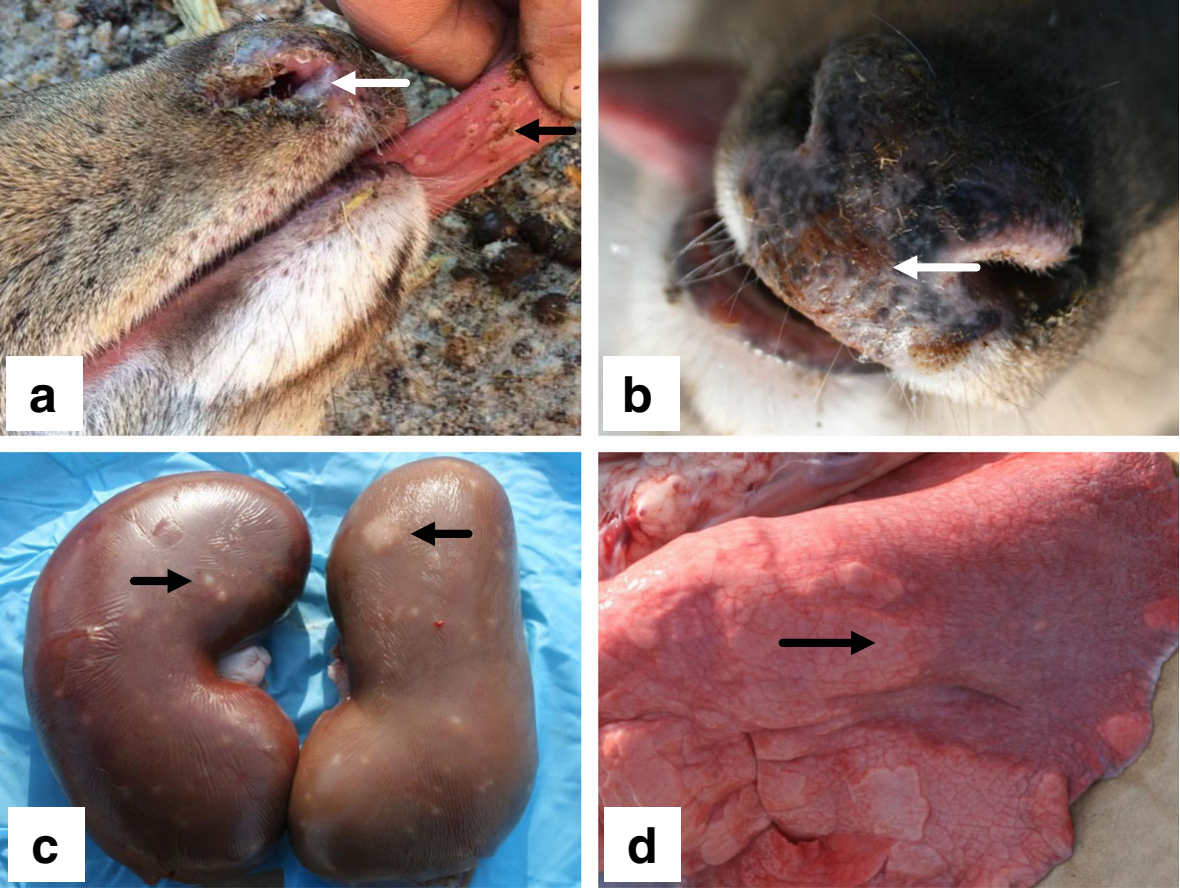
Main clinical signs and gross lesion of the third affected hind. Nasal discharge (white arrow), oral vesicles on tongue (black arrow) and mild scabs on the nose (white arrow) of the affected deer are indicated in **a** and **b**. Gross lesions on the kidney and lung are indicated in **c** and **d** respectively

In December 2016, an outbreak of a deadly disease in a captive sika deer herd was reported by a local veterinarian in Siping City, Jilin Province, China. The deer herd consisted of 186 sika deer raised in group-housing sheds. Deer of different ages and sex were grouped separately in different sheds. Each shed had a roof and its own approximately 200 m^2^ yard. Adjacent sheds were separated with bars and brickwork. In this outbreak, 3 deer hind were affected, and they were housed in two adjacent sheds. Clinical signs included high fever (> 41 °C) followed by nasal discharge. Oral vesicles developed on the tongue for each hind (Fig. 1a). Mild scabs appeared on nose of the third affected deer (Fig. 1b). In addition, all three hinds showed moderate lameness, however, no interdigital cleft vesicle, foot rot or hoof deformities could be observed. No dermal inflammation or alopecia was found. Two hinds died on the 5th and 7th day of clinical signs and were disposed of by the farm owner with only one blood sample taken. The third hind was then euthanized for postmortem examination on the 6th day when the condition deteriorated.

Main gross lesions included slight swelling of the extensor tendon and flexor tendon near the joint of hoofs, and enlargement of the kidneys and mesenteric lymph nodes. Notably, multiple pale necrotic foci approximately 0.3 cm in diameter were present on both kidneys (Fig. 1d). In addition, hemorrhagic areas appeared on the surface of the lungs (Fig. 1c).

**Fig. 2:**
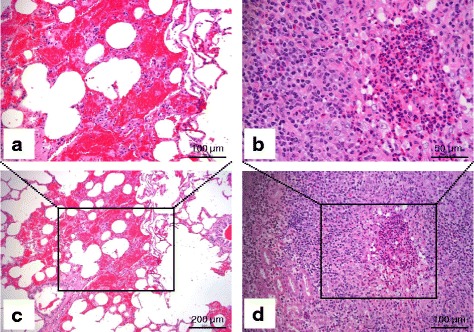
Histopathological findings. Sika deer. Lung and kidney. Lung: multifocal hemorrhage with a small amount of lymphoid cell infiltration (**a** and **c**). Kidney: large quantity of lymphoid cells, mainly lymphocytes and neutrophils, accumulated and infiltrated in the renal parenchyma. Inherent structures were replaced by the infiltrating lymphoid cells (**b** and **d**). H&E. Scales are indicated over the bars

**Fig. 3:**
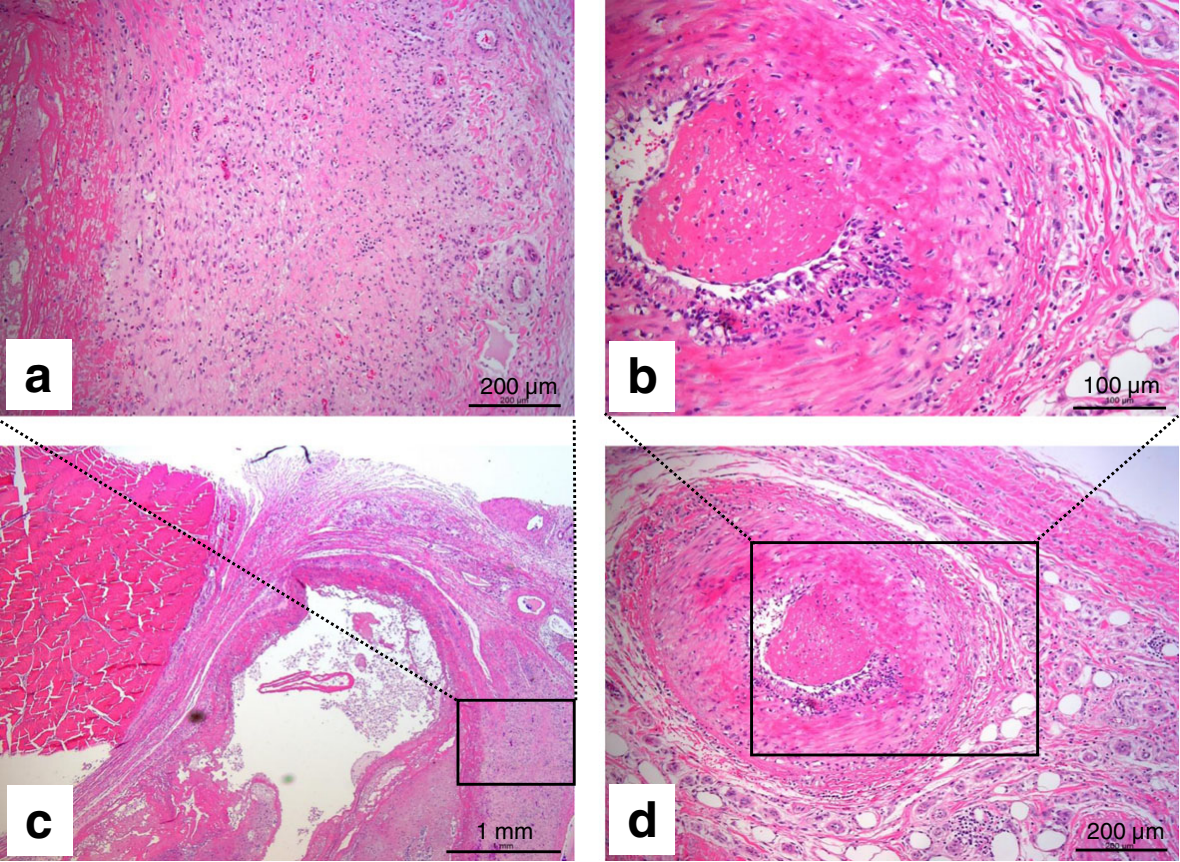
Histopathological findings. Sika deer. Tendon and surrounding subcutaneous tissue. A wide range of necrosis, lymphoid cell infiltration and fibroblast proliferation in subcutaneous tissue (**a** and **c**). Arteritis and thrombosis in subcutaneous tissue (**b** and **d**). H&E. Scales are indicated over the bars

Histopathological examination also revealed more severe lesions of the lung, kidney, heart valves and tendon. More specifically, in the lung, multifocal hemorrhages and obvious interstitial fibrosis with a small number of infiltrating lymphoid cells appeared in pulmonary tissue (Fig. 2a, c and Additional file 1: Figure S2F). In the affected kidney, large numbers of lymphoid cells, mainly lymphocytes and neutrophils, accumulated and infiltrated in the renal parenchyma, resulting in the disappearance of the inherent structure of the tissue, which was replaced by the infiltrating lymphoid cells (Fig. 2b and d). Cardiac muscle was not affected; however, extensive valvular tissue degeneration and necrosis, inflammatory infiltration and severe hemorrhage were noticed in the heart valves (Additional file 1: Figure S2E). No lesion was found in the tendon (Fig. 3c). However, in the subcutaneous tissue surrounding the tendon, a wide range of necrosis, lymphoid cell infiltration and fibroblast proliferation occurred (Fig. 3a). In addition, arteritis caused by infiltration of lymphoid cells in arterial walls in the subcutaneous tissue could be observed, and thrombosis was occasionally seen in the arteries (Fig. 3a–d). Other mild lesions included hemorrhage, lymphoid cell infiltration and/or necrosis in the liver (Additional file 1: Figure S2B), adrenal glands (Additional file 1: Figure S2D), tongue (Additional file 1: Figure S2C) and salivary glands (not shown). Lymphocytes were decreased in the lymph nodule of mesenteric lymph node (Additional file 1: Figure S2A). No lesions were found in the brain, spleen, derma and muscle. The lesions described above were in most cases suggestive of MCF.

For etiological agent identification, initial diagnostic attempts including virus isolation and viral RNA detection for foot and mouth disease virus (FMDV), peste des petits ruminants virus, epizootic hemorrhagic disease virus, bluetongue virus and vesicular stomatitis virus failed to identify any virus. Pathogenic bacterial infection was also ruled out by 16S rDNA detection. For MCFVs detection, EDTA-anticoagulated blood samples were collected from the two hinds (SP-1 and SP-2) followed by peripheral blood lymphocyte (PBL) isolation. DNA polymerase (DPOL) fragments were amplified from PBLs using a pan-herpesviral nested PCR with five degenerate primers as described previously (Additional file 2: Figure S1A). Presence of CpHV-2 in both samples was subsequently confirmed by amplicons sequencing and phylogenetic analysis (Additional file 2: Figure S1B). The resultant sequences were then submitted to the GenBank database under the accession numbers of KY475595 and MF318872. In a retrospective questionnaire for reservoir hosts, the deer farm owner claimed a well-fenced feedlot with approximately 30 domestic goats was approximately 1.0 km away in the north. Eight goats were then tested for the presence of CpHV-2 DNA in blood samples. Three were determined to be positive for the virus. Sequence alignment suggested that CpHV-2 from these goats shared 100% DPOL sequence identity with those from sika deer (data not shown).ac.

## Discussion and conclusion

To the best of our knowledge, this is the first confirmed case of CpHV-2-induced MCF, although the disease has probably long existed in China. MCF is sporadic, easily neglected and easily confused with other diseases with similar clinical signs such as foot and mouth disease or enterotoxemia. In this outbreak of MCF, the clinical signs of oral vesicles and lameness could be confused with FMD, which, however, could be ruled out by evaluating the following facts. First, epidemiologically, FMDV is a rapidly spread virus with high infectivity and transmissibility [23]. More affected deer would be expected with an FMD outbreak. Additionally, clinically, lameness in the affected deer was not due to vesicles on the interdigital cleft, but to inflammatory lesions of subcutaneous tissue. Since MCF is a systemic disease, any organ could be affected, therefore, it should be included in diagnostic considerations when an infectious disease occurs in sika deer herds.

The CpHV-2-associated MCF described in this outbreak is significantly different from MCFs in sika deer from the US and UK with respect to disease course, clinical signs and histopathological lesions. First, the hinds in Florida and in Arizona underwent chronic disease courses of ten days, one month and six months respectively, except for one deer euthanized due to seizures [17, 18]. In addition, the sika deer in the UK also experienced a two-month history of weight loss and crusting [22], while hinds in this outbreak underwent acute courses of five to seven days. Second, previously described sika deer infected with CpHV-2-associated MCF usually developed mild clinical signs of dermatitis, crusting and generalized alopecia and weight loss in most cases. However, in our case description, hinds showed no signs of weight loss or dermatitis. They displayed not only the typical MCF signs of high fever and nasal discharge but also the rarely occurring sign of lameness. Third, gross and histopathological lesions suggest the virus in this report caused more extensive inflammation and more severe hemorrhage in the lung, kidney, heart valve and liver. Whether these apparent differences were due to pathogenic characteristics of the viruses or to host aspects is yet to be elucidated.

There are many factors that affect clinical duration, clinical severity and even tissue tropism in a single MCF outbreak. Clinical signs in the mucosa and subcutaneous are most common. Lameness is rarely seen in both SA-MCF and WA-MCF. In contrast, in this MCF outbreak, we noticed the clinical sign of lameness in all affected deer while unaffected deer showed no signs of this kind. Further histological lesions in the subcutaneous tissue around a tendon correlated with clinical manifestations. In our observation, the lameness of the affected deer was most likely caused by necrosis, fibroblast proliferation and arteritis of the subcutaneous tissue. These lesions are all related to lymphoid cell infiltration, which is typical in MCF infection. No observable lesions in the brain were recorded for the euthanized deer, thus, CNS dysfunction did not contribute to the lameness in this MCF outbreak. Accordingly, we noticed that the affected deer appeared to suffer from pain in the feet rather than clinical ataxia.

It is convincing that goats near the deer farm were the natural reservoir responsible for the outbreak of MCF in sika deer, due to the identical sequence polymorphism of the detected viral DNAs. The infection route, however, was quite intriguing. The affected deer were raised in an intensive management system. They had no chance of direct contact or feed cross contamination with the reservoir goats. We thus tend to advocate an airborne infection route here. Previous studies have demonstrated that sheep shed large quantities of cell-free OvHV-2 virions via nasal secretions. Experimental infection of sheep and rabbits with ovine herpesvirus 2 via aerosolization of nasal secretions over a short distance have also been established [24–26]. In another report, Li et al. described a long distance (> 5.0 km) spread of OvHV-2 from feedlot lambs to ranch bison, causing MCF [27]. In this report, we observed that transmission of CpHV-2 from goats via aerosolization could reach and establish efficient infection at a distance of at least 1.0 km. In this scenario, 1.0 km is far from a safe distance for sika deer to prevent CpHV-2 associated MCF. However, we could not rule out the possibilities of CpHV-2 transmission through harmful fauna, given the proximity between the farms, as has been described for other viruses [28]. Wild rodents, ticks or bats could serve as vectors in this situation.

This case reminds us again of the potential risk of cross-species transmission of MCFVs, as different species demonstrate various susceptibilities to the viruses. One species carries its own sets of viruses, which may be well adapted to the original host, causing asymptomatic infection, but may bear unknown risks for another species. Therefore, keeping a safe distance from reservoirs is an effective way to avoid MCF. In addition, different deer species should be raised separately as a precaution, as MCF-like disease has been reported in sika deer co-raised with fallow deer in Inner Mongolia (personal communication with a veterinarian in a red deer farm).

In conclusion, the CpHV-2-associated MCF in this outbreak presented differently than previously described, with a more chronic, rather than acute presentation regarding disease course and severity. The domestic goat was the reservoir of the CpHV-2 which caused outbreak of the disease. The disease was transmitted at least 1.0 km in distance probably via an aerosol route. Necrosis, fibroblast proliferation and arteritis in subcutaneous tissue surrounding a tendon were the cause of lameness in this outbreak. The causative agent, CpHV-2, should be put into a differential diagnostic list when similar disease occurs in sika deer herds. Since there is no cure or vaccine for the disease, prophylactic measures should be taken accordingly. Keeping a safe distance from reservoirs is an effective approach to avoid MCF.

## Additional files

Additional file 1: Figure S2. Histopathological findings. Sika deer. Mesenteric lymph node, liver, tongue, adrenal glands, heart valve and lung. Lymphocytes decreased in the lymph nodule of a mesenteric lymph node (**Figure S2A.**) Hemorrhage, necrosis and slight lymphoid cell infiltration in the liver (**Figure S2B.**) Slight lymphoid cell infiltration in the lamina propria of the tongue (**Figure S2C.**) Focal hemorrhage in the cortex of adrenal glands (**Figure S2D**). Necrosis, inflammatory infiltration and severe hemorrhage in a heart valve (**Figure S2E.**) Interstitial fibrosis with slight lymphoid cell infiltration in the lung (**Figure S2F.**) H&E. Bar = 100 μm. (PDF 667 kb)

Additional file 2: Figure S1. Pan-herpesvirus detection and DPOL phylogeny of the isolate from affected hinds. Ethidium bromide-stained agarose gel of two amplicons from SP-1 and SP-2 respectively, using the consensus PCR assay targeting herpesviral DNA polymerase (DPOL) (**Figure S1A.**) Based on the resultant DPOL sequences, a phylogenic tree was constructed using the PhyML software (version 3.0) with LG substitution model (**Figure S1B.**) Approximate likelihood ratio test (aLRT) was performed and indicated in the node. aLRT values less than 0.50 were collapsed. Scale bar indicates 0.1 amino acid substitutions per site. DPOL GenBank accession number is AAC59454 for outgroup Bovine gammaherpesvirus 4 (BoHV-4); NC_002531 for Alcelaphine herpesvirus 1(AlHV-1); AAO88177 for MCFV-WTD; ADY17131 for Ovine gammaherpesvirus 2 (OvHV-2); APG30119 for Muskox rhadinovirus 1 (Muskox-LHV); ADY17115 for Caprine gammaherpesvirus 2 (CpHV-2); KY475595 for isolate SP-1. (PDF 129 kb)

## Abbreviations

AlHV-2: Alcelaphine herpesvirus 2
CNS: Central nervous system
CpHV-2: Caprine herpesvirus 2
DPOL: DNA polymerase
FMDV: Foot and mouth disease virus
MCF: Malignant catarrhal fever
MCFVs: Malignant catarrhal fever viruses
OvHV-2: Ovine herpesvirus 2
PBL: Peripheral blood lymphocyte
SA-MCF: Sheep-associated MCF
WA-MCF: Wildebeest-associated MCF
WTD: White-tailed deer
