# Supplementary figures and images for: Caprine herpesvirus 2-associated malignant catarrhal fever of captive sika deer (Cervus nippon) in an intensive management system
Source: BMC Vet Res. 2018 Feb 1;14:38. doi: 10.1186/s12917-018-1365-8 (PMC5796589; doi:10.1186/s12917-018-1365-8)

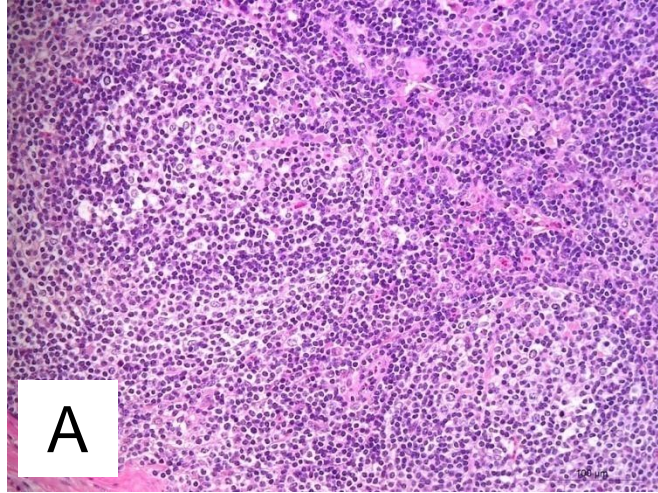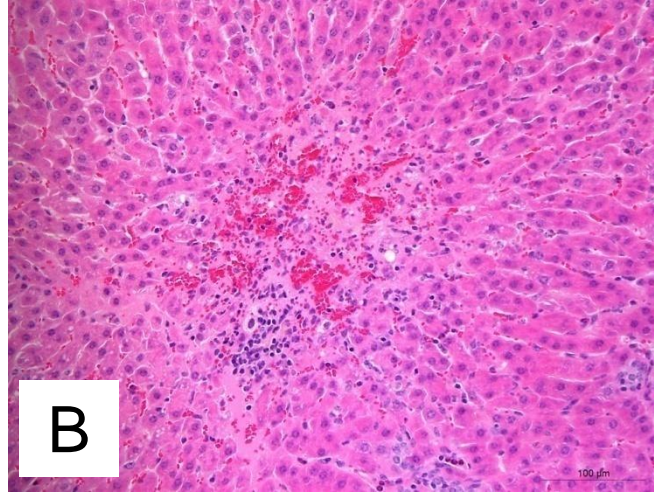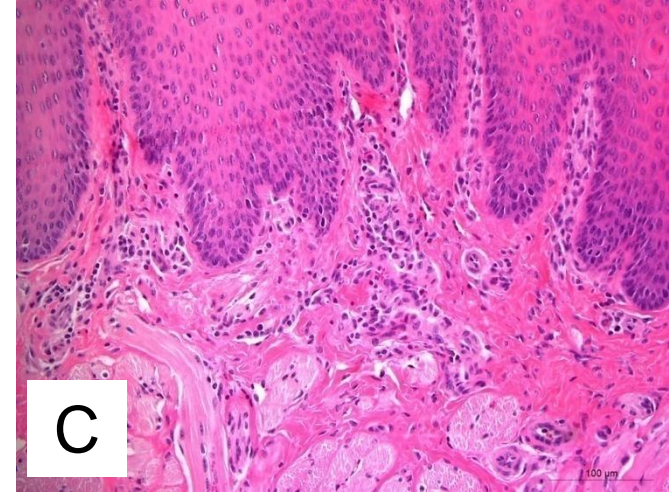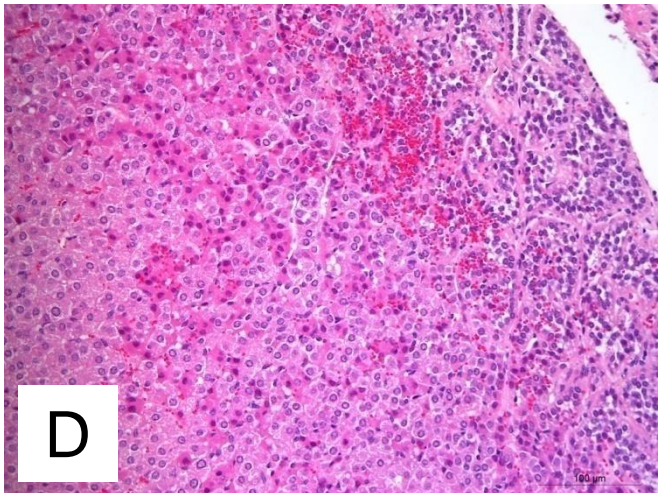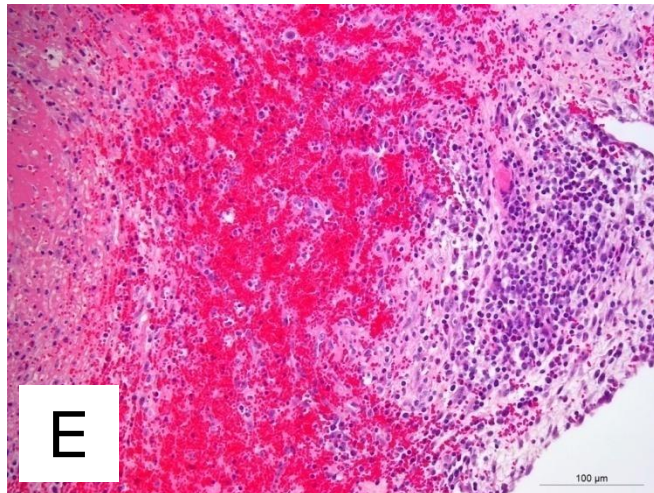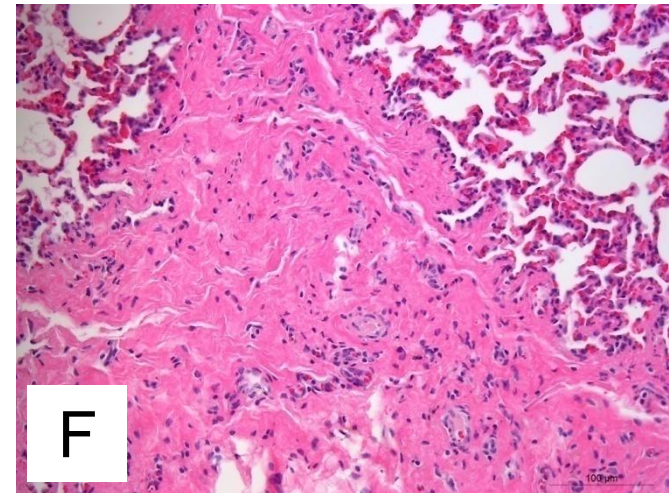

Supplement: Supplementary file 1 — Histopathological findings. Sika deer. Mesenteric lymph node, liver, tongue, adrenal glands, heart valve and lung. Lymphocytes decreased in the lymph nodule of a mesenteric lymph node (Figure S2A.) Hemorrhage, necrosis and slight lymphoid cell infiltration in the liver (Figure S2B.) Slight lymphoid cell infiltration in the lamina propria of the tongue (Figure S2C.) Focal hemorrhage in the cortex of adrenal glands (Figure S2D). Necrosis, inflammatory infiltration and severe hemorrhage in a heart valve (Figure S2E.) Interstitial fibrosis with slight lymphoid cell infiltration in the lung (Figure S2F.) H&E. Bar = 100 μm. (PDF 667 kb) [file 12917_2018_1365_MOESM1_ESM.pdf]

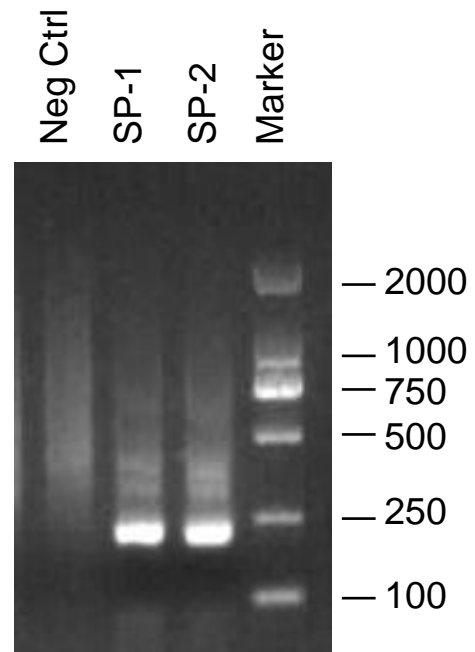

**A**

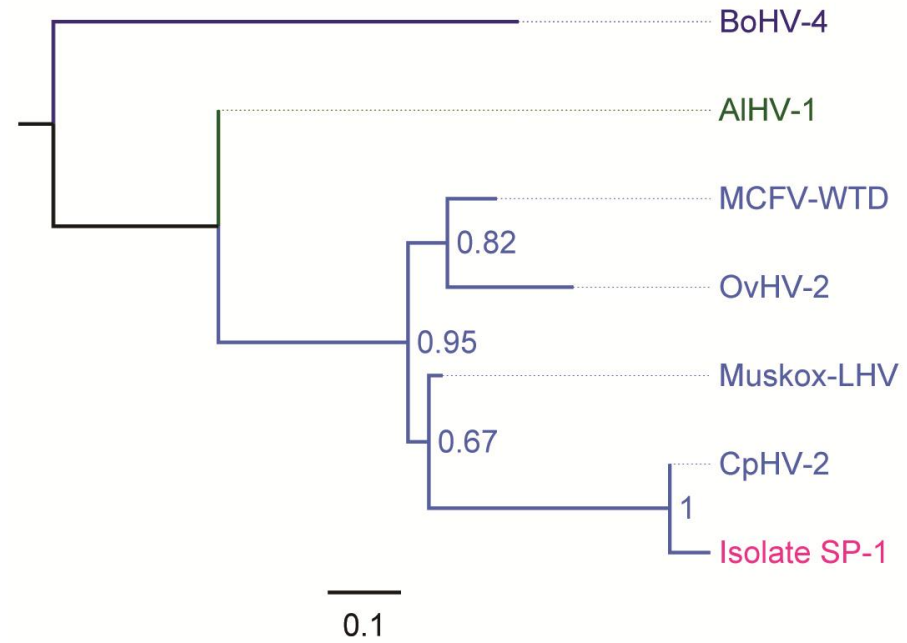

**B**

Supplement: Supplementary file 2 — Pan-herpesvirus detection and DPOL phylogeny of the isolate from affected hinds. Ethidium bromide-stained agarose gel of two amplicons from SP-1 and SP-2 respectively, using the consensus PCR assay targeting herpesviral DNA polymerase (DPOL) (Figure S1A.) Based on the resultant DPOL sequences, a phylogenic tree was constructed using the PhyML software (version 3.0) with LG substitution model (Figure S1B.) Approximate likelihood ratio test (aLRT) was performed and indicated in the node. aLRT values less than 0.50 were collapsed. Scale bar indicates 0.1 amino acid substitutions per site. DPOL GenBank accession number is AAC59454 for outgroup Bovine gammaherpesvirus 4 (BoHV-4); NC_002531 for Alcelaphine herpesvirus 1(AlHV-1); AAO88177 for MCFV-WTD; ADY17131 for Ovine gammaherpesvirus 2 (OvHV-2); APG30119 for Muskox rhadinovirus 1 (Muskox-LHV); ADY17115 for Caprine gammaherpesvirus 2 (CpHV-2); KY475595 for isolate SP-1. (PDF 129 kb) [file 12917_2018_1365_MOESM2_ESM.pdf]
